# Factors Associated with Perceived Discrimination in Healthcare Among Middle-Aged and Older Adults

**DOI:** 10.21203/rs.3.rs-6507515/v1

**Published:** 2025-05-02

**Authors:** Michael D. Green, Heather R. Farmer, Hanzhang Xu, Radha Dhingra, Qing Yang, Roland J. Thorpe, LáShauntá M. Glover, Matthew E. Dupre

**Affiliations:** Duke University School of Medicine; University of Delaware; Duke University School of Nursing; Duke University School of Medicine; Duke University School of Nursing; Johns Hopkins Bloomberg School of Public Health; Duke University School of Medicine; Duke University School of Medicine

**Keywords:** Perceived Discrimination, Healthcare Discrimination, Aging, Racial Disparities, Health Equity, Healthcare Services, Quality Improvement

## Abstract

**Background:**

Discrimination in healthcare settings impedes quality care, leading to poorer health outcomes.

**Objective:**

To examine racial differences in perceived discrimination in healthcare settings across age among middle-aged and older adults and identify factors associated with these experiences.

**Design:**

Longitudinal cohort data from the Health and Retirement Study collected between 2008 and 2020.

**Participants:**

The sample included 17,478 United States adults aged 50 and older who had at least one doctor visit or hospitalization in the prior two years.

**Main Measures:**

Self-reported perceived discrimination in healthcare settings, measured using an item from the Everyday Discrimination Scale and categorized as “never” versus “ever” experienced discrimination. Generalized linear mixed models were used to identify factors associated with experiencing discrimination. Assessed factors included sociodemographic (age, gender, marital status, education, wealth, insurance status, employment) and clinical characteristics (depressive symptoms, difficulty with activities of daily living [ADLs], number of doctor visits, hospitalizations, body mass index [BMI], and comorbidities).

**Results:**

Black adults were significantly more likely to experience discrimination in healthcare settings than White adults, and these disparities were most pronounced at younger ages. Factors associated with higher odds of reporting discrimination included Black race, male gender, not being married, being uninsured, higher educational attainment, depressive symptoms, difficulty with ADLs, history of arthritis, and higher BMI. In race-stratified analyses, unemployment was associated with higher odds of reporting discrimination among Black adults. Among White adults, being unmarried and uninsured were significant factors associated with discrimination.

**Conclusions and Relevance:**

Black adults reported higher rates of perceived discrimination in healthcare settings than White adults, especially during middle adulthood. Multiple sociodemographic and clinical factors were associated with these experiences. These findings underscore the need to address discrimination in healthcare to improve patient-provider relationships among middle-aged and older adults.

## Introduction

Discrimination within healthcare settings is a critical barrier to high quality care, impacting patient health outcomes and contributing to negative patient experiences.^1–4^ Prior research has demonstrated that patients who experience discrimination receive suboptimal care and are more likely to be non-adherent to treatment regimens.^5–11^ This in turn worsens health disparities and inflates long-term healthcare expenditures.^12–14^

Discrimination in healthcare is a distinct exposure separate from general forms of discrimination that does not have an identified location of exposure. In clinical settings, patients’ unique sociodemographic, physical, and health-related characteristics (e.g., age, body weight) may lead to variations in lived experiences which consequently shapes their exposure to differential treatment,^15^ alongside clinical factors which have documented racial differences in adverse outcomes.^16^ Recent research demonstrates that more than one third of adults report experiencing some form of discrimination in healthcare settings.^17^ However, studies have not examined the factors associated with perceived discrimination in healthcare settings uniquely among middle-aged and older adults. Understanding which sociodemographic characteristics, such as age, gender, and socioeconomic status, as well as whether certain chronic conditions and healthcare-related factors are associated with perceived discrimination in healthcare settings is particularly critical in later adulthood. Patients with different sociodemographic and clinical profiles may be treated differently, through conscious or unconscious bias,^18^ which in turn can affect their help seeking behaviors, treatment decisions, and quality of treatment they receive.^12,19,20^ This is especially important during middle-age and older adulthood, a period marked by increased interactions with healthcare systems, and a population who is likely subject to greater impacts by the quality of those interactions.^21,22^

Discrimination experiences among middle-aged and older adults are critical to understanding health inequities.^23^ Discrimination’s impacts are present throughout life course, where many older adults in America who faced discrimination in early life course (e.g. being denied loans, freedom to use facilities) are currently facing consequences of that discrimination (delayed retirement, age discrimination in workplace, long-term psychosocial stress).^24–26^ Prior studies have investigated racial disparities in health systems, and found that Black adults living in the U.S. generally report more distrust in the healthcare system as well as more frequent experiences of discrimination.^9,27^ However, it remains unclear whether these disparities persist specifically among older adults, a group with heightened healthcare needs and vulnerability to the impacts of systemic racism.^28–30^

To address this critical knowledge gap, our objectives are twofold: first, to document racial differences in perceived discrimination in healthcare settings among middle-aged and older adults across age and second, to examine the factors associated with these reported experiences in Black and White middle-aged and older adults.

## Methods

### Data source:

Our analysis used data from the Health and Retirement Study (HRS), the largest ongoing nationally-representative longitudinal study of U.S. adults over age 50. The HRS is sponsored by the National Institute on Aging (grant number NIA U01AG009740) and is conducted by the University of Michigan.^31^ The HRS has accumulated over three decades of data on more than 40,000 individuals since its launch in 1992. Comprehensive details on its survey methodology and response rates are documented elsewhere.^32^ Beginning in 2006, the HRS selected a random half-sample of respondents to collect detailed psychosocial data every four years.^33,34^ Subsequent data were collected in the 2008 random half-sample and continued through 2020, providing quadrennial follow-up data for all participants.

The current study included respondents aged 50 years and older who participated in the HRS Psychosocial and Lifestyle Questionnaire administered from 2008 to 2020 (n = 21,887). We excluded respondents identifying race as “other” due to insufficient detail in this category (n = 1,862). In addition, participants who reported no doctor visits or hospitalizations at baseline were not included (n = 1,364) because they did not report recent interactions with doctors or hospitals. We also excluded individuals who did not respond to the question for perceived discrimination in a healthcare setting (n = 341) described below. Finally, we excluded individuals with missing data for any of the covariates at baseline (n = 842). Individuals with missing data were presented in Supplementary Table 2. Overall, missing data was low among study participants.

The final analytic sample included 17,478 participants (n = 14,134 White adults; n = 3,344 Black adults) who were followed for up to 12 years over the study period. The study was approved by the Duke University Health System Institutional Review Board (Pro00108869).

### Discrimination in Healthcare Settings

Our primary outcome was perceived discrimination in a healthcare setting. The measure was obtained from the Everyday Discrimination Scale (EDS),^35^ which consists of six items designed to measure the frequency of discrimination experienced in various daily contexts, including interactions in retail environments, restaurants, and wider social contexts. In 2008, the EDS in the HRS was updated to capture discrimination experienced in healthcare settings. This item asks, “In your day-to-day life how often have any of the following things happened to you: You receive poorer service or treatment than other people from doctors or hospitals.” Our preliminary analyses found that there was limited variability in the frequency of experiencing discrimination in healthcare settings (see Supplementary Table 1). We considered study baseline, the first time that they provided a response to this question. For the current analysis, we dichotomized the responses “never” as 0 for “never experienced discrimination” and 1 for “any experienced discrimination” (“less than once a year”, “a few times a year”, “a few times a month”, “at least once a week”, “almost every day”). This approach was chosen to capture the distinction between individuals who never encountered discrimination in healthcare settings and those who did, regardless of the frequency or intensity of their experiences.^36^ This measure is recorded every 4 years through the HRS Psychosocial and Lifestyle Questionnaire.

### Covariates

*Sociodemographic* factors included age (years), gender (female or male), Hispanic ethnicity (y/n), race (White or Black) marital status (currently married/partnered or not), educational attainment (years), total household wealth based on assets per household member (log-transformed), insurance status (uninsured or insured), and employment status (working, not working, or retired). *Clinical* factors included depressive symptoms assessed by a 8-item Center for Epidemiologic Studies Depression (CES-D) scale (count; 0–8), difficulty with activities of daily living ([ADLs]; count; 0–6),^37^ number of doctor visits in the past 2 years (count; winsorized at 50+), number of hospitalizations in the past 2 years (count; winsorized at 5+), body mass index ([BMI]; count), and self-reported doctor diagnoses of high blood pressure, diabetes, lung disease, heart disease, stroke, cancer, and arthritis. Gender, race, and ethnicity were time-constant from baseline and the remaining variables are time-varying. We also included a time-constant indicator for mortality to account for attrition due to death during the study follow-up, following previous studies.^38,39^

### Analytical approach

We first assessed baseline differences between those who “never” and “any “reported discrimination in healthcare separately for Black and White adults using Mann-Whitney tests for continuous/ordinal variables and chi-squared tests for binary/categorical variables. We then used generalized linear mixed models (with a logit link function) to examine the longitudinal changes in reported discrimination in healthcare settings by race across age. This approach allowed us to account for individuals’ repeated observations (level 1) nested within individuals (level 2). Initial analyses assessed different polynomial functions of age (i.e., linear, quadratic, and cubic) based on Bayesian Information Criterion and indicated that a linear function provided best fit for the parameterization of age. We then included race, ethnicity, an interaction term between race and age, and an adjustment for mortality attrition in the model. Predicted probabilities derived from this model were used to illustrate racial differences in age-related trajectories of reported discrimination in a healthcare setting.

Next, we examined which factors were associated with the likelihood of experiencing discrimination in healthcare settings in the overall sample and by White and Black adults separately. To do this, we included a wide range of sociodemographic and clinical factors in the generalized linear mixed models for the full sample and then tested interactions between race and the sociodemographic and clinical covariates. Finally, we stratified the analyses into White adults and Black adults to demonstrate the factors separately by race. All models tested for intercept and slope (i.e., age interactions) differences in the associations. Results are reported as odds ratios (OR) with 95% confidence intervals (CI), with *P* < 0.050 considered to be statistically significant. Statistical analyses were conducted in Stata 18; figures were constructed in RStudio Version 2023.12.1.402.

## Results

Table 1 presents the baseline characteristics of White and Black adults in the overall sample and by reported discrimination in healthcare settings. Overall, a higher proportion of Black adults (23.50%) reported discrimination in healthcare settings than White adults (17.50%) at baseline. For both Black and White adults, lower household wealth, being uninsured, depressive symptoms, difficulty in ADLs, more frequent doctor visits, higher BMI, and a history of arthritis were associated with reported discrimination in a healthcare setting at baseline.

**Figure 1:**
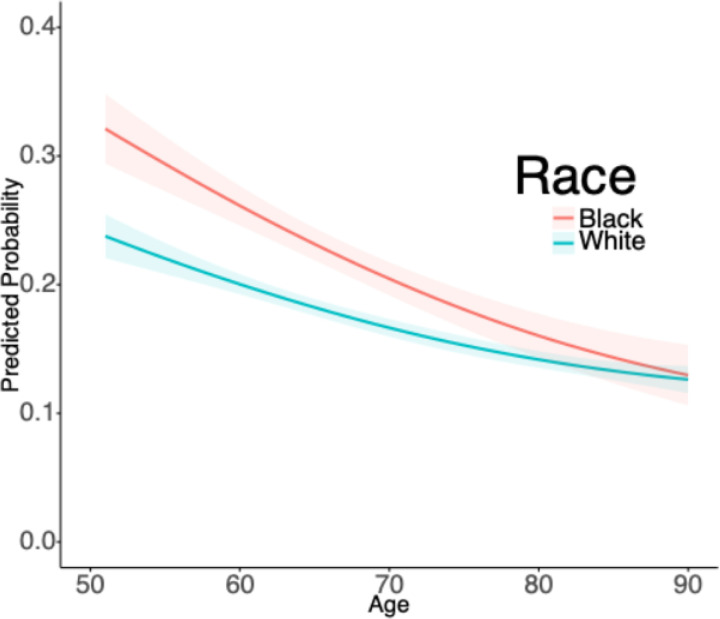
Predicted Probabilities of Reporting Discrimination in Healthcare Settings Among U.S. Middle-Aged and Older Adults, Health and Retirement Study (2008–2020) Note: Plots are based on mixed models with indicators for Black race (P<0.001), interactions with time (race*age; P=0.013), Hispanic ethnicity (P=0.713), mortality over the study period (P<0.001), male gender (P=0.002). Shaded areas represent 95% confidence intervals.

Figure 1 illustrates the predicted probabilities from the mixed models for the age-related association between race and experiencing discrimination in a healthcare setting. Overall, the results showed that discrimination in a healthcare setting was more often reported at younger ages than at older ages. We also found that Black adults were significantly more likely to experience discrimination in healthcare settings than White adults (*P* < 0.001), and that these disparities were most pronounced at younger ages (race*age interaction: *P* = 0.013). By later life (ages > 80), there were no significant racial differences in the predicted probability of reporting discrimination in a healthcare setting.

**Figure 2:**
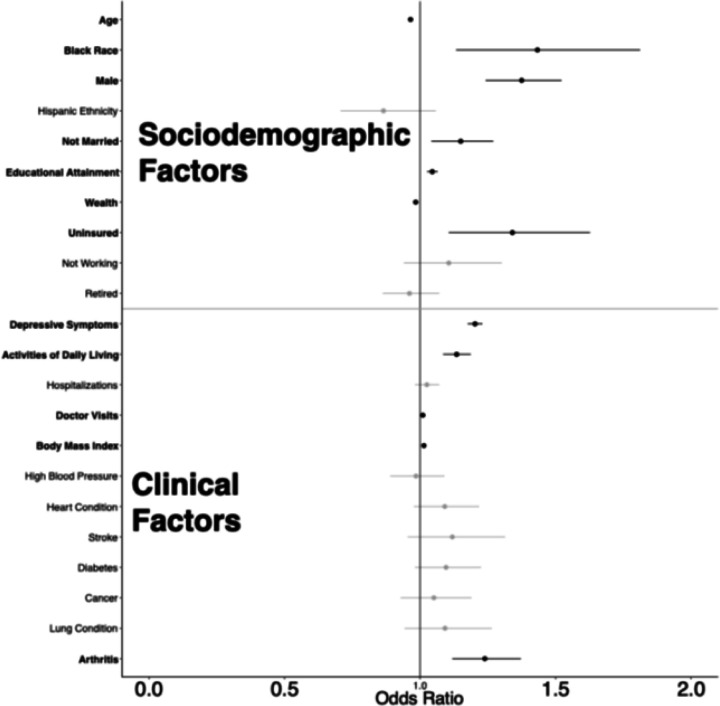
Adjusted Odds Ratios (95% Confidence Intervals) for the Factors Associated with Reporting Discrimination in Healthcare Settings Among U.S. Middle-aged and Older Adults, Health and Retirement Study (2008–2020) Note: Statistically significant values (P < 0.05) are bolded in black. Wealth variable log transformed. Time constant mortality to account for attrition, and an interaction between Black race and age were components of the model.

Figure 2 presents results from the generalized linear mixed models, which produced adjusted odds ratios for sociodemographic and clinical factors associated with recent discrimination in healthcare settings for all adults in the overall sample. Sociodemographic factors associated with increased odds of experiencing discrimination in healthcare included Black race (OR = 1.43, 95% CI [1.13–1.81]), male gender (OR = 1.37, CI [1.24–1.52]), not being married (OR = 1.15, CI [1.04–1.27]), and being uninsured (OR = 1.34, CI [1.11–1.63]). More years of educational attainment showed a positive association (OR = 1.05, CI [1.03–1.07]), while wealth demonstrated a negative association (OR = 0.98, CI [0.97–0.99]). Clinical factors revealed multiple significant associations with healthcare discrimination, including increased depressive symptoms (OR = 1.20, CI [1.18–1.23]), greater difficulty with Activities of Daily Living (OR = 1.13, CI [1.08–1.19]), more doctor visits (OR = 1.01, CI [1.01–1.01]), history of arthritis (OR = 1.24, CI [1.12–1.37]), and higher body mass index (OR = 1.01, CI [1.01–1.02]). Notably, when an interaction term for Black race was introduced, several significant differences emerged, specifically unemployment (P = 0.001), ADL limitations (P = 0.022), and a history of arthritis (P = 0.050).

**Figure 3:**
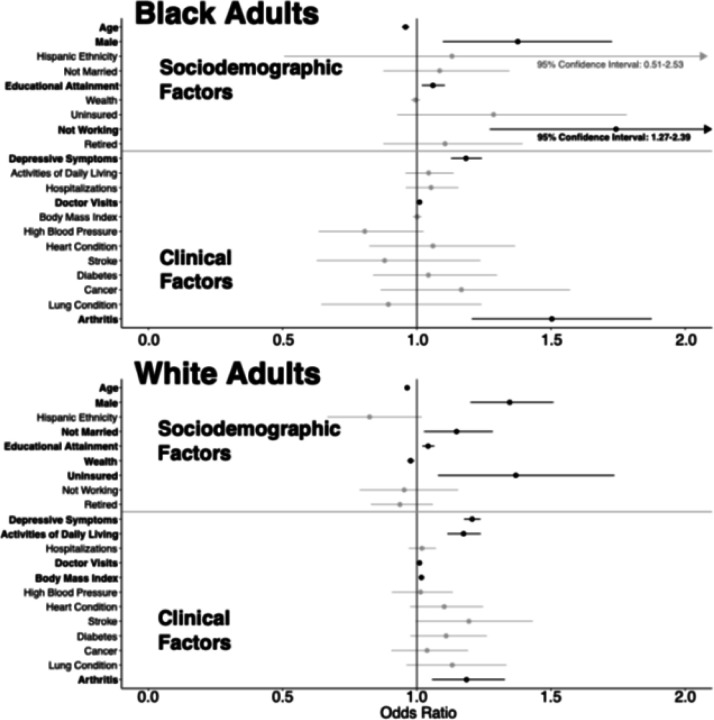
Adjusted Odds Ratios (95% Confidence Intervals) for the Factors Associated with Reporting Discrimination in Healthcare Settings Among Black and White U.S. Middle-aged and Older Adults, Health and Retirement Study (2008–2020) Note: Statistically significant values (P < 0.05) are bolded in black. Wealth variable log transformed. Time-constant mortality was a component of each model to account for attrition.

Figure 3 illustrates the factors associated with discrimination in healthcare settings for Black and White adults separately, using generalized linear mixed models to produce adjusted ORs for all model variables. Among White adults, all factors from the overall sample remained significant. In contrast, for Black adults, there were many differences between the overall sample. Not being married, wealth, higher BMI, ADLs, and being uninsured were no longer significantly associated with experiencing discrimination in healthcare settings. Unemployment emerged as a new factor significantly associated with a higher likelihood of experiencing discrimination (OR = 1.74, CI [1.27–2.39]). Both Black and White adults had significantly increased odd for reporting discrimination with a history of arthritis, higher education, depressive symptoms, male gender, and more doctor visits when examined separate.

When testing the interaction between age and our covariates, we identified that for White adults there were significant slope differences across increasing age for being uninsured (P = 0.015), decreasing age while having a higher BMI (P < 0.001), and decreasing age with a history of hypertension (P = 0.044). There were not significant age-based slope differences for Black adults.

## Discussion

Our study was the first to identify the sociodemographic and clinical characteristics of patients that are associated with reported discrimination over time, measured by age. This study showed that Black adults were significantly more likely to report discrimination in healthcare settings compared to White adults. With increasing age, however, these disparities diminished. Our findings make an important contribution to existing literature by demonstrating the longitudinal dynamics of reported discrimination in healthcare settings in a diverse sample of middle-aged and older U.S. adults. Our analysis also identified several factors that were associated with the likelihood of reporting discrimination in healthcare settings, with worse health status and difficulties in physical functioning emerging as a recurrent theme.

Our findings suggest that middle-aged adults are more likely to experience discrimination in healthcare environments relative to older adults. Moreover, we found that the racial disparity in reported discrimination is most pronounced in those at younger ages (i.e., middle adulthood). These findings generally align with previous research highlighting the prevalence of race-based and socioeconomic status-based discrimination among younger adults.^40,41^ The reasons for this are not entirely clear. For example, it is possible that the observed differences across age reflect potential cohort differences—given the U.S. historical context where older Black adults have lived through periods of legal segregation and the direct consequences of this unequal treatment.^42,43^ Additional research is needed to better understand the factors contributing to these patterns.

When considering sociodemographic, and clinical factors collectively, White and Black adults both showed a similar pattern of associations with regard to male gender, higher educational attainment, and adverse health-related status (e.g., history of arthritis and depressive symptoms) being associated with a greater likelihood of reporting discrimination. Despite these significant factors shared by Black and White adults, there were a few differences, as well. In particular, difficulty with ADLs was incrementally associated with experiencing discrimination in a healthcare setting for White adults, but not for Black adults. Previous studies using HRS data have also linked discrimination in healthcare settings to disability and worsening functional limitations.^44,45^ Also notably, our analysis also suggests that increased frequency of doctor visits was associated with higher likelihood of experiencing perceived discrimination. Prior research has demonstrated a link between higher physician interaction, reported discrimination, and increased likelihood to delay care. ^44^Taken together, it is important to further disentangle the temporal order of whether discrimination leads to a decreased desire to see physicians and/or if more doctor visits increase the risk of experiencing discrimination.

A previous study has examined racial differences in health-related discrimination experiences, linking discrimination in healthcare to elevated cardiovascular biomarkers and including race and ethnicity as covariates.^46^ Our research, however, stratifies by race, providing a detailed analysis of how these associations manifest across different racial groups. Among White adults, lower wealth, being uninsured, and being unmarried were associated with experiencing discrimination in healthcare settings. Conversely, among Black adults, unemployment, was a factor associated with experiencing discrimination in healthcare settings. Older adults who are unemployed (but not retired) may face unique challenges, such as limited access to healthcare resources. Consequently, if these individuals disproportionately report experiencing discrimination, this finding is particularly concerning and warrants attention.

Racial inequities exist across a large range of health-related outcomes (e.g., readmissions, hypertension).^47,48^ However, these inequities are not a random occurrence: structural racism has been widely acknowledged as a driver of inequities, in that it has a long history in the United States that has built in, fostered and normalized discrimination across multiple institutions (e.g., healthcare, employment) and continues to do so in present day.^49^ Moreover, structural racism in conjunction with interpersonal discrimination is linked to disproportionately greater health consequences across the life course.^50–52^

Some limitations should be considered when interpreting our results. First, the racial categories available for analysis were limited. Future research should broaden the racial categories beyond those used here to enrich our understanding of discrimination across different groups. Second, although our measure of discrimination was informative because it is attributed to a specific location, we could not determine the reasons why an individual felt discriminated against (e.g., age, weight, race, socioeconomic status) because it is not distinguishable among the individual items of the EDS. Other studies have identified common patterns for reports of discrimination across multiple settings, and identifying any differences in discrimination in healthcare would be an important addition to our findings.^53,54^ This measure also was utilized as a dichotomous variable, opposed to an ordinal one which can capture variation in severity of discrimination experiences. Third, health status and clinical comorbidities were ascertained with self-reported measures that were not adjudicated. Interestingly, past research has shown that Black adults reporting racial discrimination tend to exhibit higher initial cognitive function; whereas White adults tend to have lower cognitive function.^55^ Future studies with electronic health records or more frequent patient-reported outcomes could reduce potential recall bias, enhance clinical diagnosis accuracy, and allow for an overall more robust analysis of the association between discrimination and health status. Concerns surrounding accuracy and meaning of self-reported measures should also be considered for perceived discrimination. For example, individuals might face explicit mistreatment (e.g. care that does not align with clinical guidelines), but they are unaware of this mistreatment. Educational attainment has previously been connected to a higher likelihood of experiencing discrimination in general settings.^10,56^ Our study identifies that more educated populations also experience more discrimination in healthcare settings, as well. This is important in the context that either people who are more highly educated are either experiencing more discrimination, or, potentially, that individuals with less education are less aware of discrimination. Perceptions of discrimination should be analyzed with more refined clinical context to understand how those experiences align with the treatment that the patient has received, in order to understand the most appropriate corrective action to achieve better quality care. Finally, future studies with shorter intervals between consecutive surveys capturing reported discrimination, more detailed measures of discrimination, and a wider array of social determinants of health (*e.g.*, access to healthcare services and social support) could also yield valuable insights into the multifaceted influences on healthcare discrimination. Addressing these gaps can better inform targeted interventions (e.g. anti-bias training in medical education) and policies to improve healthcare equity across all demographics.

Discrimination in healthcare is a detrimental experience that providers, and healthcare researchers can address though trainings in: allyship, bias literacy, and emotional regulation.^57^ Our research illuminates the association of discrimination in healthcare settings with multiple facets of an individual’s lived experience. The sociodemographic and clinical factors identified in this work provide much-needed insight into patient-level characteristics that likely have significance in clinical settings which could be important to consider when constructing a plan for combating discrimination in healthcare and analyzing the adverse impact it will have on population health. This study provides an important first step to help address discrimination in healthcare settings and promote equitable care for all individuals.

## Supplementary Material

Supplement 1

## Figures and Tables

**Table 1:** Baseline Characteristics of Middle-Aged and Older Adults by Race and Experiences of Discrimination in a Healthcare Setting. Health and Retirement Study 2008–2020

	White Adults				Black Adults			
	Total	Never	Any	*P-value*	Total	Never	Any	*P-value*
	N = 14,134	N = 11,660	N = 2,474		N = 3,344	N = 2,558	N = 786	
Sociodemographic Factors								
Age	66.92 (10.74)	67.32 (10.73)	65.03 (10.54)	< .001	62.67 (9.23)	63.33 (9.47)	60.55 (8.04)	< .001
Male	5,953 (42.12%)	4,850 (41.60%)	1,103 (44.58%)	.006	1,182 (35.89%)	900 (35.57%)	282 (36.90%)	.499
Hispanic Ethnicity	1,200 (8.49%)	983 (8.43%)	217 (8.77%)	.581	53 (1.67%)	38 (1.52%)	2.16%	.223
Unmarried	5,120 (36.22%)	4,155 (35.63%)	965 (39.01%)	.002	1,941 (58.61%)	1,473 (58.13%)	468 (60.18%)	.308
Educational Attainment	13.13 (2.90)	13.13 (2.89)	13.17 (2.94)	.138	12.61 (2.84)	12.55 (2.81)	12.79 (2.93)	.006
Total Household Wealth	$310k ($640k)	$320k ($670k)	$250k ($480k)	< .001	$64k ($130k)	$65k ($130k)	$60k ($130k)	.012
Uninsured	675 (4.78%)	509 (4.37%)	166 (6.71%)	< .001	309 (9.24%)	219 (8.56%)	90 (11.45%)	.014
Employment Status				.001				.728
Working	5,738 (40.60%)	4,678 (40.12%)	1,060 (42.85%)		1,435 (42.91%)	1,116 (43.63%)	319 (40.59%)	
Not Working	1,364 (9.65%)	1,090 (9.35%)	274 (11.08%)		397 (11.87%)	260 (10.16%)	137 (17.43%)	
Retired	7,032 (49.75%)	5,892 (50.53%)	1,140 (46.08%)		1,512 (45.22%)	1,182 (46.21%)	330 (41.98%)	
Clinical Factors								
Depressive Symptoms	1.34 (1.93)	1.21 (1.80)	1.97 (2.34)	< .001	1.83 (2.11)	1.67 (1.98)	2.38 (2.41)	< .001
ADLS	0.31 (0.89)	0.27 (0.84)	0.47 (1.07)	< .001	0.49 (1.16)	0.44 (1.09)	0.65 (1.34)	< .001
Doctor Visits	9.59 (10.27)	9.30 (9.96)	10.93 (11.53)	< .001	9.39 (10.68)	8.94 (10.14)	10.87 (12.18)	.001
Hospitalizations	0.46 (0.93)	0.44 (0.91)	0.53 (1.03)	< .001	0.50 (1.02)	0.48 (0.97)	0.59 (1.16)	.208
Body Mass Index	28.42 (5.93)	28.21 (5.80)	29.40 (6.43)	< .001	30.69 (7.02)	30.55 (6.95)	31.16 (7.21)	.013
High Blood Pressure	7,722 (54.63%)	6,338 (54.36%)	1,384 (55.94%)	.150	2,416 (73.36%)	1,876 (74.24%)	540 (70.48%)	.037
Heart Condition	3,223 (22.80%)	2,624 (22.50%)	599 (24.21%)	.066	661 (19.83%)	502 (19.70%)	159 (20.23%)	.746
Stroke	1,082 (7.66%)	847 (7.26%)	235 (9.50%)	< .001	318 (9.51%)	249 (9.73%)	69 (8.78%)	.424
Diabetes	2,722 (19.26%)	2,165 (18.57%)	557 (22.51%)	< .001	984 (29.81%)	742 (29.36%)	242 (31.30%)	.299
Cancer	2,132 (15.08%)	1,742 (14.94%)	390 (15.76%)	.298	348 (10.53%)	260 (10.24%)	88 (11.45%)	.334
Lung Condition	1,394 (9.86%)	1,098 (9.42%)	296 (11.96%)	< .001	269 (8.10%)	207 (8.13%)	62 (8.02%)	.917
Arthritis	8,025 (56.78%)	6,557 (56.23%)	1,468 (59.34%)	.005	1,844 (55.71%)	1,379 (54.38%)	465 (60.05%)	.005
Died during the study period	3,947 (28.32%)	3,258 (28.31%)	689 (28.36%)	.926	693 (20.84%)	549 (21.54%)	144 (18.58%)	.073

Note: Continuous variables are reported as means with standard deviations in parenthesis. Binary and ordinal variables are reported as counts (unless under 25) with percentages in parenthesis. Total household wealth rounded to nearest thousands, and reported by thousand. Statistical significance was determined with Mann Whitney tests for continuous/ordinal variables, and Chi-Squared tests for binary variables at an alpha of 0.05. *Abbreviations*: ADL= Activities of daily living
