# Supplementary material for: Factors Associated with Perceived Discrimination in Healthcare Among Middle-Aged and Older Adults
Source: Res Sq. 2025 May 2:rs.3.rs-6507515. Preprint. [Version 1] doi: 10.21203/rs.3.rs-6507515/v1 (PMC12060977; doi:10.21203/rs.3.rs-6507515/v1)
Supplement: Supplement 1 [file NIHPPRS6507515v1-supplement-1.pdf]

Supplementary Files

This is a list of supplementary files associated with this preprint. Click to download.

- ResearchSquareRevision1SupplementaryFile.docx
